# Molecular Characterization of Circulating Yellow Fever Viruses from Outbreak in Ghana, 2021–2022

**DOI:** 10.3201/eid2909.221671

**Published:** 2023-09

**Authors:** Joseph Humphrey Kofi Bonney, Terrel Sanders, Deborah Pratt, Bright Agbodzi, Dennis Laryea, Nana Kwame Fredua Agyeman, Selassie Kumordjie, Keren Attiku, Patience Lartekai Adams, Gideon Aning Boateng, Sally-Ann Ohene, Christopher Tamal, Gifty Mawuli, Clara Yeboah, Samuel Dadzie, Chrysantus Kubio, Franklin Asiedu-Bekoe, John Kofi Odoom

**Affiliations:** Noguchi Memorial Institute for Medical Research Virology Department, Legon Accra, Ghana (J.H.K. Bonney, D. Pratt, K. Attiku, P.L. Adams, G.A. Boateng, G. Mawuli, J.K. Odoom);; US Naval Medical Research Unit, No. 3, Ghana Detachment, Accra, Ghana (T. Sanders, B. Agbodzi, S. Kumordjie, C. Yeboah);; Ghana Health Service Public Health Unit, Accra (D. Laryea, N.K.F. Agyeman, F. Asiedu-Bekoe);; World Health Organization Ghana Country Office, Accra (S.-A. Ohene, C. Tamal);; Noguchi Memorial Institute for Medical Research Department of Parasitology, Legon Accra (S. Dadzie);; Ghana Health Service–Savannah Regional Health Directorate, Damongo, Ghana (C. Kubio)

**Keywords:** yellow fever, yellow fever virus, viruses, vector-borne infections, viral hemorrhagic diseases, whole-genome sequencing, molecular characterization, Ghana

## Abstract

Yellow fever virus, transmitted by infected *Aedes* spp. mosquitoes, causes an acute viral hemorrhagic disease. During October 2021–February 2022, a yellow fever outbreak in some communities in Ghana resulted in 70 confirmed cases with 35 deaths (case-fatality rate 50%). The outbreak started in a predominantly unvaccinated nomadic community in the Savannah region, from which 65% of the cases came. The molecular amplification methods we used for diagnosis produced full-length DNA sequences from 3 confirmed cases. Phylogenetic analysis characterized the 3 sequences within West Africa genotype II; strains shared a close homology with sequences from Cote d’Ivoire and Senegal. We deployed more sensitive advanced molecular diagnostic techniques, which enabled earlier detection, helped control spread, and improved case management. We urge increased efforts from health authorities to vaccinate vulnerable groups in difficult-to-access areas and to educate the population about potential risks for yellow fever infections.

Yellow fever virus (YFV), transmitted by infected *Aedes* spp. mosquitoes, causes a viral hemorrhagic disease, typically acute, with case-fatality rates up to 50%. The disease remains a major public health problem, especially in West Africa, where outbreaks occur every year. In Ghana, yellow fever outbreaks have been observed in 5-year cycles over the past 20 years. However, increased recorded incidence and death during these outbreaks can be partially attributed to improved diagnostic efforts from laboratory investigations. 

Initial influenza-like signs and symptoms from yellow fever typically improve within 5 days; however, 15%–25% of infected persons progress to complications, including liver damage, which increases risk for bleeding and kidney problems (*1*). YFV (strain Asibi), a mosquito-borne flavivirus, was first isolated in 1927 from a patient in Ghana (*2*). Despite having an effective vaccine, 17D strain, with >500 million doses administered to humans (*3*), YFV infection remains a public health threat in certain regions of the world (*1*); ≈1 billion persons are estimated to live in regions endemic for yellow fever. In 2013 alone, YFV caused ≈127,000 severe infections and 45,000 deaths globally (*1*); ≈90% of deaths occur in Africa (*4*). 

Yellow fever has been endemic in Ghana since it was first documented (*5*). Major outbreaks have occurred, notably in the 1970s and 1980s (*6*). One recent outbreak, which occurred in the West Gonja district in the Savannah region of Ghana in 2015, resulted in 3 deaths from 12 confirmed cases (*7*). Additional sporadic cases have been rumored or confirmed since the 2015 outbreak. 

Little is known about the genetic diversity and evolutionary dynamics of YFV, mainly because few genomic sequences from wild virus isolates have been identified. For this outbreak investigation, we aimed to use molecular assays to rapidly detect and confirm or disprove presence of YFV among case-patients. We also sought to characterize virus strains in clinical specimens from YFV-positive case-patients from the most affected communities to discover the molecular epidemiology of the outbreak within the identified regions. The institutional review board of the Noguchi Memorial Institute for Medical Research (NMIMR) approved experimental protocols for molecular detection of viral hemorrhagic fevers (VHFs), including YFV (NMIMR-IRB-003/07-08). 

## Materials and Methods 

### Setting and Study Design 

**Figure 1:**
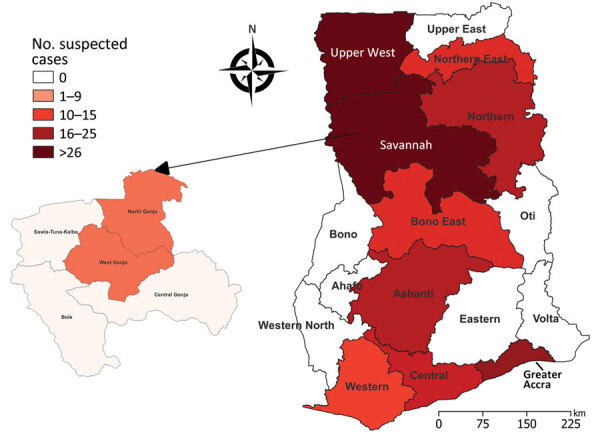
Distribution of suspected yellow fever cases among the 16 regions of Ghana, January 2021–February 2022. Callout map at left shows the high-incidence Savannah region containing 2 districts, North and West Gonja, that had the highest numbers of cases among the 6 districts in the region. Map created using QGIS version 3.26.1-Buenos Aires (https://qgis.org); Ghana boundary coordinates obtained from the Ghana Statistical Service.

Elevated yellow fever incidence during October 2021–February 2022 led to an outbreak being declared in Ghana. We collected clinical specimens of serum from patients in health facilities in the outbreak areas, predominantly Damongo, Busunu, and Kawankura communities in West Gonja district and Daboya and Kagbal communities in North Gonja district, which constitute 2/6 districts of the Savannah region in Ghana (Figure 1). We collected additional specimens from health facilities in adjoining districts and regions, including Sawla-Tuna-Kalba district and Bono East region. We submitted 188 clinical specimens from patients with suspected YFV to NMIMR for molecular diagnosis. Nucleic acid amplification testing of the specimens confirmed 70 yellow fever cases from communities in 4 regions (Savannah, Upper West, Bono, and Oti) in northern Ghana. Because of 35 recorded deaths and a case-fatality ratio of 50%, public health interventions were swiftly initiated among the nomadic populations most affected. Those populations live in forested areas, including in the immediate vicinity of a forest reserve in the Savannah region. We placed all patients with suspected yellow fever based on case definitions in isolation or holding rooms and used requisite infection prevention and control precautions to manage cases. Public health and laboratory staff using appropriate personal protective equipment collected clinical specimens and recorded demographic and health history information, including age, sex, travel history, vaccination status, date of hospital admission, and residential location. We sent the 188 clinical specimens taken during the outbreak period to laboratories for further investigation, including characterizing virus strains. Age range of case-patients was 4 months to 70 years; most exhibited signs/symptoms such as body pain, fever, abdominal pain, vomiting, jaundice, and bleeding from the gums. Slightly more case-patients were male (54%) than female (46%). 

### Background Observations 

**Figure 2:**
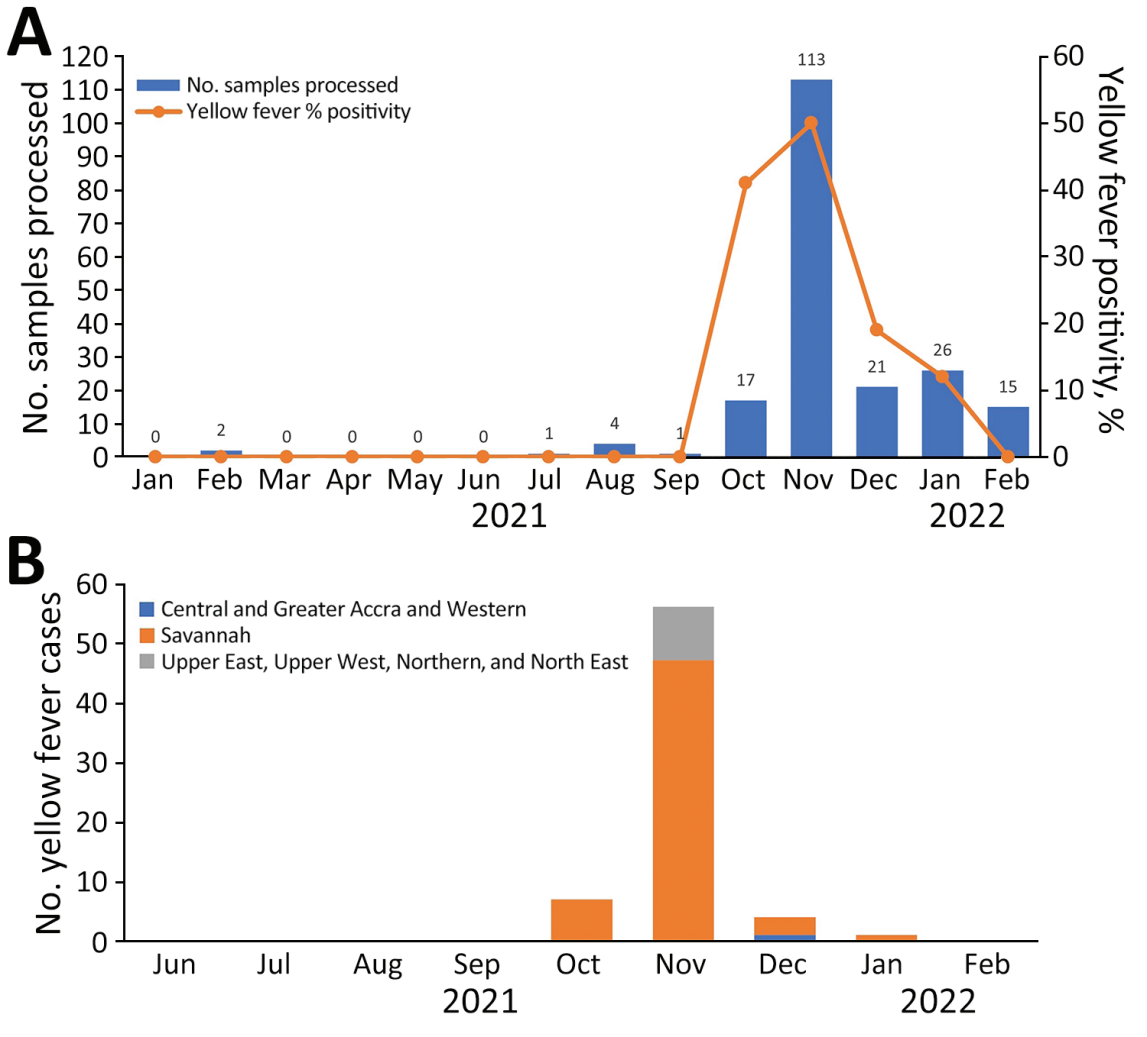
Distribution of yellow fever cases over time, Ghana, January 2021–February 2022. A) Percentage positivity over the outbreak period and total number of samples processed. B) Numbers of yellow fever–positive participants over the outbreak period within the 3 regional categories.

An activity for passive surveillance of VHFs, established in 2016 in response to the 2014–2016 Ebola virus disease outbreak in some West Africa countries, provided routine reports on suspected yellow fever cases submitted from health facilities (*8*). From that surveillance activity, 12 suspected cases were reported, and the patients were screened. All 12 reports were submitted during February–September 2021, before the YFV outbreak began, and patients tested negative for all VHFs on the panel of viruses (Table 1): Ebola, Marburg, Lassa, dengue, chikungunya, and yellow fever (Figure 2). 

**Table 1:** Details of PCR testing and sequence analysis from study of yellow fever in Ghana, 2021–2022*

Virus	Reagent kit	Cycles	Primer sequences, 5′ → 3′	Target gene	Amplicon length, bp
Lassa virus	QIAGEN OneStep RT-PCR	45	36E2:ACCGGGGATCCTAGGCATTT	5′ UTR/GPC	320
			LVS-339-rev:GTTCTTTGTGCAGGAMAGGGGCATKGTCAT		
YFV	QIAGEN/Ambion OneStep rRT-PCR	45	RF:AAATCCTGKGTGCTAATTGAGGTGYATTGG		
			RR:ACATDWTCTGGTCARTTCTCTGCTAATCGC		
			RProbe: gCAAATCgAgTTgCTAggCAATAAACACATT[BHQdT]g[THF]A [FAMdT] TAATTTTRATCgTTC -Ph		
Filovirus	QIAGEN Filo OneStep RT-PCR	45	FiloA2.2:AAGCCTTTCCTAGCAACATGATGGT	L	290
			FiloA2.3:AAGCATTCCCTAGCAACATGATGGT		
			FiloA2.4:AAGCATTTCCTAGCAATATGATGGT		
			FiloA2.4:AAGCATTTCCTAGCAATATGATGGT		
			Filo B-Ra:GTGAGGAGGGCTATAAAAGTCACTGACATG		
Trioplex (*12*)					
Dengue	Invitrogen Superscript III Platinum OneStep qRT-PCR	45	NA	C	171
CHIKV				E1	208
Zika				NS5	209

*QIAGEN, http://www.qiagen.com; Invitrogen, Thermo Fisher, https://www.thermofisher.com. CHIKV, chikungunya virus; RT-PCR, reverse transcription PCR; qRT-PCR, quantitative RT-PCR; YFV, yellow fever virus.

### Real-Time Reverse Transcription PCR Assays

We extracted viral nucleic acid from 140 μL of serum using the QIAamp viral RNA kit (QIAGEN, https://www.qiagen.com). We performed all PCR assays in 25 μL of Master Mix with 2.5 μL or 5 μL nucleic acid extract as a template (Table 1). We used real-time reverse transcription PCR (rRT-PCR) for Lassa virus (*9*), YFV (*10*), and filoviruses including Ebola and Marburg viruses (*10*,*11*) and a Trioplex rRT-PCR (*12*) for qualitative detection and differentiation of dengue, chikungunya, and Zika virus RNA in the clinical specimens taken from the suspected case-patients. We performed amplifications using the Applied Biosystems 7500 Fast/Standard Dx Real-Time PCR instrument (ThermoFisher Scientific, https://www.thermofisher.com).

### Trioplex rRT-PCR 

The Trioplex assay, designed for research purposes only (*12*), was created to test simultaneously for the presence of dengue, chikungunya, and Zika viruses using primers and dual-labeled probes and a reverse transcription step to produce copy DNA (cDNA) from RNA in the sample. The probe binds to the target DNA between the 2 unlabeled PCR primers. During the PCR extension process, the polymerase extends the unlabeled primers using the template strand as a guide. The rRT-PCR instrumentation detects fluorescence; with each successive PCR cycle, fluorescence increases in proportion to the amount of target nucleic acid present. This assay identifies Zika, chikungunya, and dengue virus RNA during the acute phase of infection and up to 14 days after onset of signs/symptoms (*12*).

### Whole Genome Sequencing

We prepared sequencing libraries using Illumina DNA prep with enrichment (Illumina, https://www.illumina.com), according to the manufacturer’s instructions. We performed viral enrichment using custom target capture probes (Twist Bioscience, https://www.twistbioscience.com). We fragmented the extracted RNA, spiked it with mosquito RNA, and reverse-transcribed it to cDNA. We achieved dual indexing of cDNA libraries using IDT unique dual indexes (Integrated DNA Technologies, https://www.idtdna.com). We enriched libraries by using the 1-plex pooling strategy following a protocol described elsewhere (*13*). We sequenced barcoded pooled libraries on an Illumina MiSeq with version 3 reagent kits. 

### Sequence Analysis

We quality filtered demultiplexed raw fastq files to Phred scores ≥20, filtered them for minimum read length of 20 bp, and adaptor trimmed them using BBDuk (decontamination using kmers; https://sourceforge.net/projects/bbmap). We confirmed read quality using FastQC tool (https://sourceforge.net/projects/fastqc.mirror). We used the resultant high-quality reads for de novo assembly using the SPAdes assembler version 3.15.2 (https://github.com/ablab/spades) (*14*). We used the largest contig from the de novo assembly to query the nonredundant nucleotide database (GenBank) to obtain the best matching reference sequence. We employed the retrieved reference for reference-based assembly using Bowtie2 (https://bowtie-bio.sourceforge.net/bowtie2/index.shtml) (*15*). To make a consensus call, we required >3 times read-depth coverage; we treated positions lacking this depth of coverage as missing (labeled N). 

### Phylogenetic Analysis

We submitted consensus sequences from the final assemblies to the Genome Detective virus tool (https://www.genomedetective.com) for genotyping. For phylogenetic analysis, we selected complete genomes covering the 4 major YFV genotypes in addition to our strains. We conducted genome alignment using MUSCLE (https://www.ebi.ac.uk/Tools/msa/muscle) and phylogenetic construction using MEGAX software (*16*,*17*). To correct for the effects of ambiguous alignments because of polymorphisms in the 5′ and 3′ untranslated regions, we trimmed the sequences to the open reading frames (ORFs) and conducted all subsequent phylogenetic analyses on the ORFs. We conducted maximum likelihood phylogenetic analysis on the sequences using the generalized time reversible plus gamma distribution substitution model, which was inferred as the best fit model for the data in MEGAX. We ascertained the robustness of each node of the phylogenetic tree using the bootstrap method with 1,000 replicates. We used FigTree version 1.4.4 (http://tree.bio.ed.ac.uk/software/figtree) for tree visualization and annotation. 

### Accession Numbers

We attempted to sequence all PCR-confirmed positive samples from the outbreak. However, only 3 positive samples yielded DNA sequencing data of sufficiently good quality to be sequenced on the Illumina next-generation sequencing platform. We deposited those sequences into GenBank (accession nos. OM066735–37). 

## Results 

The outbreak lasted from mid-October 2021 through the first week of February 2022; a total of 188 clinical specimens of whole blood serum or plasma were submitted for testing within that period. We submitted one half-portion of each sample from suspected case-patients within the identified outbreak regions (Figure 1) to the virology department of NMIMR, a World Health Organization–recognized laboratory, for molecular confirmation of yellow fever (*18*). We sent the other half-portion to the National Public Health Reference Laboratory (NPHRL) in Accra, Ghana, for serologic testing for YFV IgM. After ruling out dengue, West Nile, and Zika viral infections by differential diagnosis (*18*), YFV-positive samples were forwarded to the WHO-designated regional reference laboratory in Dakar, Senegal. 

We determined suspected yellow fever cases on the basis of location in high-incidence regions and signs/symptoms associated with YFV infection: muscle and joint pain, abdominal pain, difficulty swallowing, difficulty breathing, hiccups, loss of appetite, skin rash, anorexia, myalgia, dizziness, malaise, agitation, swollen buttocks, convulsion, chills, runny nose, chest pain, cough, and lethargy. Yellow fever was less common in the Central, Greater Accra, and Western regions than the Savannah region (odds ratio [OR] 0.08, 95% CI 0.01–0.63) (Table 2) and more common among persons who exhibited signs/symptoms (OR 2.03, 95% CI 1.11–3.71; p = 0.022) (Table 2) than those who did not. During the outbreak, we observed the highest number of confirmed cases in November 2021 (Figure 2). 

**Table 2:** Distributions of patient sex, region, and signs/symptoms in study of yellow fever in Ghana, 2021–2022

Characteristics	Odds ratio (95% CI)	p value
Sex		
M	Referent	0.079
F	0.58 (0.31–1.07)	
Region		
Savannah	Referent	0.024
Central, Greater Accra, Western	0.08 (0.01–0.63)	
Upper East, Upper West, Northern, North East	0.53 (0.23–1.23)	
Ashanti, Bono East		
Signs/symptoms		
Fever	1.68 (0.75–3.75)	0.207
Jaundice	0.6 (0.1–3.75)	0.584
Hemorrhage	3.17 (0.89–11.24)	0.074
Other*	2.03 (1.11–3.71)	0.022

*Muscle and joint pain, abdominal pain, difficulty swallowing, difficulty breathing, hiccups, loss of appetite, skin rash, anorexia, myalgia, dizziness, malaise, agitation, swollen buttocks, convulsions, chills, runny nose, chest pain, cough, and lethargy

### Demographic and Virologic Findings

We performed Trioplex screening for qualitative detection and differentiation of dengue, chikungunya, and Zika viruses and RT-PCR testing for other VHFs existing in the regions, including Lassa, Ebola, and Marburg; all samples tested negative for those viruses. However, rRT-PCR testing confirmed yellow fever (Table 1) in 70/188 (37%) patients, 64% of whom were male (Table 3). Age range of all patients was 4–24 years; mean age was 7 years for YFV-negative and 11 years for YFV-positive patients (Table 3). Health facilities in 10/16 regions in Ghana, in the coastal (Central, Greater Accra, and Western), midlands (Ashanti and Bono East), and northern (Upper East, Upper West, Northern, and Northeast) areas of the country and in the Savannah region, submitted suspected cases for testing (Figure 1). The highest percentage of total (65%), positive (84%), and negative (57%) samples submitted came from the Savannah region (Table 3). Results from the Savannah region, in northwest Ghana, showed a statistically significant higher association with yellow fever relative to other regions, including >2 times as many cases as from other northern regions combined. Calculating percentages of the signs/symptoms of patients screened (Table 3) indicated fever, jaundice, and hemorrhage were the predominate clinical signs among both YFV-negative and -positive patients, although the absolute numbers were not statistically significant. 

**Table 3:** Demographics and signs/symptoms of patients in study of yellow fever in Ghana, 2021–2022

Variable	Yellow fever test results		p value
	Negative, n = 118	Positive, n = 70	
Sex			0.078
M	58 (50.4)	44 (63.8)	
F	57 (49.6)	25 (36.2)	
Median age, y (interquartile range)	7 (4–19)	11 (4–23.5)	0.172
Region			
Central, Greater Accra, Western	14 (12.2)	1 (1.4)	<0.001
Ashanti, Bono East	15 (13.0)	0	
Savannah	65 (56.5)	58 (84.1)	
Upper East, Upper West, Northern, North East	21 (18.3)	10 (14.5)	
Unknown	3	1	
Signs/symptoms			
Fever	62/88 (70.5)	44/55 (70.0)	0.205
Jaundice	3/47 (6.4)	2/51 (3.9)	0.58
Hemorrhage	4/118 (3.4)	7/70 (10.0)	0.062
Other†	39/118 (33.1)	35/70 (50.0)	0.021

*Values are no. (%) except as indicated. †Muscle and joint pain, abdominal pain, difficulty swallowing, difficulty breathing, hiccups, loss of appetite, skin rash, anorexia, myalgia, dizziness, malaise, agitation, swollen buttocks, convulsions, chills, runny nose, chest pain, cough, and lethargy

### Sequence Analysis and Phylogeny

**Figure 3:**
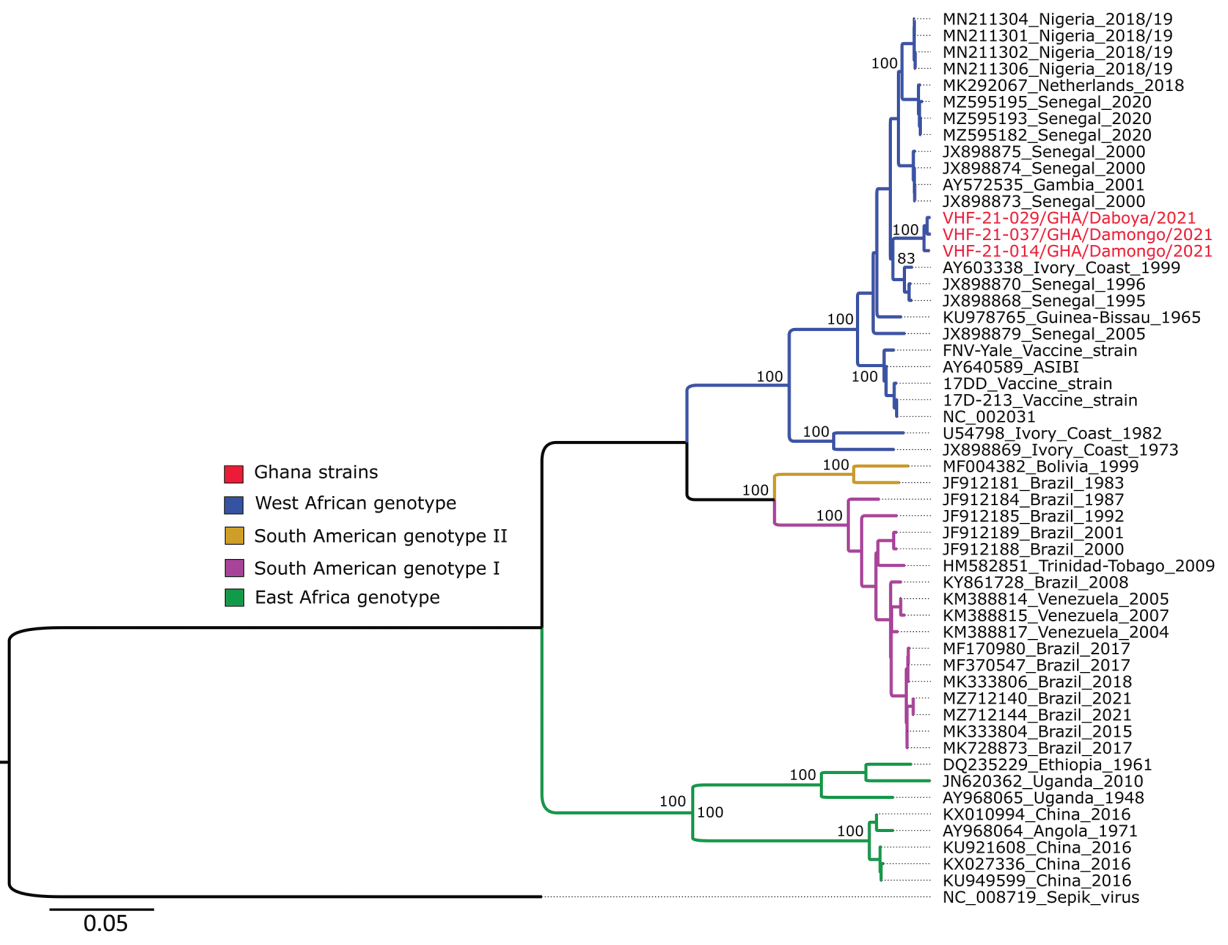
Phylogenetic analysis of yellow fever virus sequences from 3 confirmed cases in Ghana during January 2021–February 2022 (red text) compared with reference sequences obtained from GenBank in January 2022 (identified by GenBank accession number and country of origin).). Virus genotypes are indicated with different color nodes on the tree. Some branches with low support values were collapsed for clarity of presentation. Scale bar indicates substitution per site.

The Genome Detective virus tool grouped all 3 Ghana yellow fever strains within West Africa genotype II. Complete ORF maximum-likelihood phylogeny showed the 3 yellow fever strains from the outbreak area in Ghana to be closely related to each other and to sequences from Senegal and Cote d’Ivoire (Figure 3). Those sequences all clustered within West Africa genotype II, which is less heterogeneous than the other 8 known West Africa genotypes (*19*). 

## Discussion

The October 2021–February 2022 yellow fever outbreak in parts of Ghana renewed calls and highlighted the need for timely laboratory confirmation of suspected yellow fever cases as an essential part of effective responses. The greater sensitivity of advanced molecular diagnostic techniques deployed for laboratory testing during outbreak investigations distinguished those methods from previous serologic assays. The improved performance of those diagnostic techniques enabled us to characterize the circulating outbreak strains and deposit yellow fever strains from Ghana with GenBank. 

Initial outbreak cases were identified at the West Gonja District Hospital in the West Gonja municipality of the Savannah region. Three index case-patients from adjoining localities spent an average of 3 days in the hospital and died before their clinical specimens could be tested and results released. In addition to necessary laboratory confirmation, final determination of yellow fever diagnosis must be made on a case-by-case basis, in the context of clinical manifestations, epidemiology, and vaccination history (*18*,*19*). Early identification and diagnosis, leading to prompt response, are essential for successfully controlling communicable disease outbreaks and ensuring global health security. 

Implementing the Global Health Security agenda (*20*) developed by health and allied ministries in Ghana has enhanced capacity for outbreak response. Improving advanced laboratory testing capacity and establishing an advanced-level field epidemiology training program were among other core components contributing to quicker response time, reduced illness and death, and controlled risk of spread. Diagnostic specificity was ensured because the molecular methods deployed in our laboratory investigations minimized false-positive test results by targeting the specific molecule of interest. In addition, turnaround times are shorter for molecular diagnostic methods than for serologic testing, decreasing time from specimen receipt to test result reporting. 

In disease-endemic areas, outbreaks provide historic patterns or trends to help guide health workers make preliminary diagnoses and begin case management before final diagnoses are laboratory confirmed. The yellow fever outbreak documented in our study began in the Savannah region of Ghana, which also recorded the highest numbers of confirmed cases (70) and 35 deaths (case-fatality rate 50%). In retrospect, analysis of 2011 and 2015–2016 surveillance data on confirmed yellow fever in the region indicated a 5-year cycle of occurrence (*21*). A 2011 outbreak of yellow fever began in November in the Northern region (since delineated into Savannah and Northern regions) and by February 2012 had spread to 10 additional regions and led to 7 deaths (*21*). In comparison, the 2021–2022 outbreak recorded the worst death counts and rates over the intervening period. That greater severity might be because initial cases occurred among Fulani, pastoral nomads who move about in remote settlements and have substantial populations of unvaccinated youth (*22*). 

In accordance with the standard algorithm for viral detection and means of differential diagnosis, we used RT-PCRs developed for VHF-associated viruses (*10*,*11*) and multiplex assays (*12*). All 188 clinical specimens of serum or plasma received from the health facilities during the outbreak, in addition to the 12 received before the onset of the outbreak, were screened and tested negative for Lassa fever, Ebola, Marburg, dengue, chikungunya, and Zika viral infections. Those findings are consistent with a previous study in which we established that overall VHF incidence is low in Ghana and contributes little to hospital-identified morbidity (*23*). However, although yellow fever is classified as a VHF, low incidence does not extend to that disease, which is known to be endemic in Ghana. 

Using an RT-PCR assay developed to detect YFV RNA, we confirmed that 70/188 suspected case-samples submitted to NMIMR during the 2021–2022 outbreak were positive for yellow fever (*10*). More than half (102/188, 64%) of the samples received during the outbreak were from male patients. Combined with the median age of 11 years (Table 2), that finding suggests that the outbreak affected younger and working-aged men and boys engaged in nomadic pastoral lifestyles more than other demographic groups. This observation corroborates findings made in farming communities in other parts of Africa under similar outbreak conditions (*24*). Because persons seeking healthcare, especially during outbreak conditions, tend to be more severely affected, the actual number of persons with yellow fever was likely higher than the number for whom we submitted samples to NMIMR for testing; persons with cases of subclinical or mildly symptomatic yellow fever might not have been sampled, so cases might have gone undetected. Reflecting the iceberg concept, which indicates that for each detected case there is considerable potential for many more undetected infections, it has been estimated that 1 severe case of yellow fever might represent an additional 3–20 asymptomatic or mild infections (*25*). 

The highest percentages of clinical specimens—total (65%), positive (84%), and negative (57%)—came from the Savannah region (Table 3), which had case numbers >2 times those recorded from the other northern regions combined. That finding supports the assertion that the yellow fever outbreak started and peaked in the region. Past outbreaks in the region have occurred during the dry season months, October–February, as did the 2021–2022 outbreak. Water stored in containers around households provides habitat for mosquitoes and might increase their populations. In addition, an upsurge in farming activities during those periods in preparation for the rainy season might have led to more frequent exposure to mosquito vectors in remote areas. However, mosquito species trapped during outbreak investigations, including *Aedes aegypti aegypti* (2%), *Ae. aegypti formosus* (39%), and *Culex* spp. (58%), tested negative for YFV. This finding suggests either low virus density in the mosquito population sampled or the contribution of forest-dwelling mosquito species that mediate vector infection rates in sylvatic outbreaks. 

Yellow fever was commonly detected among symptomatic persons, including those exhibiting hemorrhage. Calculated percentages of patients screened indicated that fever, hemorrhage, and other signs/symptoms were predominantly observed for both negative and positive patients, although we found no statistically significant association between signs/symptoms and yellow fever detection (Table 3). Yellow fever is classified a VHF because of shared signs/symptoms with other VHFs, aside from fever among some. Patients with yellow fever often initially exhibit fever and general malaise, signs/symptoms common in other tropical diseases, including malaria and typhoid. Those similar manifestations make differentiating VHFs, including yellow fever, from other tropical diseases more difficult but vital for proper management and to curtail spread. 

The sequences generated from this outbreak investigation clustered among sequences known in literature and documented to be circulating in Ghana. Phylogenetic analysis revealed some close homology among the sequences from yellow fever–positive patient samples. Although the strains circulated in different outbreak communities, they were closely related to each other and to strains circulating in Senegal and Cote d’Ivoire; the strains all clustered within West Africa genotype II. Seven YFV genotypes have been described (*26*–*30*), 2 in South America and 5 in Africa, namely West Africa genotype I (Nigeria, Cameroon, and Gabon), West Africa genotype II (Senegal, Guinea, Ivory Coast, and Ghana), East and Central Africa genotype (Sudan, Ethiopia, Central African Republic, and Democratic Republic of Congo), East Africa genotype (Kenya), and Angola genotype (Angola). Less homogeneous outbreaks of yellow fever have been documented within areas of endemicity (*21*). Sequences of the 2 West Africa genotypes dominate in outbreaks for reasons possibly attributable to genetic variability that might affect the virulence of the virus. Sequences belonging to West Africa genotype I show more heterogeneity than West Africa II and East/Central Africa genotypes (*26*), which could indicate stronger evolutionary activity. 

In conclusion, in this yellow fever outbreak in Ghana, a more sensitive pathogen detection approach during our laboratory outbreak investigations enabled us to reduce time between the outbreak and when first cases were detected, which proved useful for reducing time between when the first cases were detected after the actual beginning of the outbreak and subsequent initiation of disease control interventions leading to more effective disease management. Rapid response is an essential component in successfully controlling infectious disease outbreaks and ensuring global health security interests. Moreover, identifying full-length sequences of 3 confirmed YFV strains provided vital genomic surveillance information about circulating strains and potential risks. On the basis of our findings, we urge increased efforts from health authorities to educate and vaccinate vulnerable groups in difficult-to-access areas to reduce potential risks for yellow fever infections. 

## References

[1] World Health Organization. Yellow fever [cited 2021 Nov 12]. https://www.who.int/news-room/fact-sheets/detail/yellow-fever

[2] Barrett ADT, Weaver SC. Arboviruses: alphaviruses, flaviviruses and bunyaviruses: encephalitis; yellow fever; dengue; haemorrhagic fever; miscellaneous tropical fevers; undifferentiated fever. In: Greenwood D, Barer M, Slack R, Irving W, editors. Medical microbiology: eighteenth edition. London: Churchill Livingstone; 2012.

[3] Gardner CL, Ryman KD. Yellow fever: a reemerging threat. Clin Lab Med. 2010;30:237–60. 10.1016/j.cll.2010.01.001 20513550 PMC4349381

[4] Tolle MA. Mosquito-borne diseases. Curr Probl Pediatr Adolesc Health Care. 2009;39:97–140. 10.1016/j.cppeds.2009.01.001 19327647

[5] Scott DE. Epidemic disease in Ghana, 1901–1960. London: Oxford University Press; 1965.

[6] Agadzi VK, Boatin BA, Appawu MA, Mingle JAA, Addy PA. Yellow fever in Ghana, 1977-80. Bull World Health Organ. 1984;62:577–83. 6333294 PMC2536325

[7] Fresh yellow fever claims 3 lives in West Gonja [cited 2022 Mar 23]. https://www.modernghana.com/news/666626/fresh-yellow-fever-outbreak-claims-3-lives-in-west-gonja.html

[8] Bonney JH, Asigbee TW, Kotey E, Attiku K, Asiedu-Bekoe F, Mawuli G, et al. Molecular detection of viral pathogens from suspected viral hemorrhagic fever patients in Ghana. Health Sciences Investigations Journal. 2020;1:31–5. 10.46829/hsijournal.2020.6.1.1.31-35

[9] Escadafal C, Faye O, Sall AA, Faye O, Weidmann M, Strohmeier O, et al. Rapid molecular assays for the detection of yellow fever virus in low-resource settings. PLoS Negl Trop Dis. 2014;8:e2730. 10.1371/journal.pntd.0002730 24603874 PMC3945292

[10] Towner JS, Rollin PE, Bausch DG, Sanchez A, Crary SM, Vincent M, et al. Rapid diagnosis of Ebola hemorrhagic fever by reverse transcription-PCR in an outbreak setting and assessment of patient viral load as a predictor of outcome. J Virol. 2004;78:4330–41. 10.1128/JVI.78.8.4330-4341.2004 15047846 PMC374287

[11] Drosten C, Göttig S, Schilling S, Asper M, Panning M, Schmitz H, et al. Rapid detection and quantification of RNA of Ebola and Marburg viruses, Lassa virus, Crimean-Congo hemorrhagic fever virus, Rift Valley fever virus, dengue virus, and yellow fever virus by real-time reverse transcription-PCR. J Clin Microbiol. 2002;40:2323–30. 10.1128/JCM.40.7.2323-2330.2002 12089242 PMC120575

[12] Santiago GA, Vázquez J, Courtney S, Matías KY, Andersen LE, Colón C, et al. Performance of the Trioplex real-time RT-PCR assay for detection of Zika, dengue, and chikungunya viruses. Nat Commun. 2018;9:1391. 10.1038/s41467-018-03772-1 29643334 PMC5895813

[13] Blackley DJ, Wiley MR, Ladner JT, Fallah M, Lo T, Gilbert ML, et al. Reduced evolutionary rate in reemerged Ebola virus transmission chains. Sci Adv. 2016;2:e1600378. 10.1126/sciadv.1600378 27386513 PMC4928956

[14] Prjibelski A, Antipov D, Meleshko D, Lapidus A, Korobeynikov A. Using SPAdes de novo assembler. Curr Protoc Bioinformatics. 2020;70:e102. 10.1002/cpbi.102 32559359

[15] Langmead B, Salzberg SL. Fast gapped-read alignment with Bowtie 2. Nat Methods. 2012;9:357–9. 10.1038/nmeth.1923 22388286 PMC3322381

[16] Kumar S, Stecher G, Li M, Knyaz C, Tamura K. MEGA X: molecular evolutionary genetics analysis across computing platforms. Mol Biol Evol. 2018;35:1547–9. 10.1093/molbev/msy096 29722887 PMC5967553

[17] Kalyaanamoorthy S, Minh BQ, Wong TKF, von Haeseler A, Jermiin LS. ModelFinder: fast model selection for accurate phylogenetic estimates. Nat Methods. 2017;14:587–9. 10.1038/nmeth.4285 28481363 PMC5453245

[18] World Health Organization. Yellow fever laboratory diagnostic testing in Africa [cited 2022 Mar 23]. https://apps.who.int/iris/bitstream/handle/10665/246226/who-ohe-yf-lab-16.1-eng.pdf

[19] Stock NK, Laraway H, Faye O, Diallo M, Niedrig M, Sall AA. Biological and phylogenetic characteristics of yellow fever virus lineages from West Africa. J Virol. 2013;87:2895–907. 10.1128/JVI.01116-12 23269797 PMC3571399

[20] Global health security agenda [cited 2022 Mar 26]. https://globalhealthsecurityagenda.org

[21] International Federation of Red Cross and Red Crescent Societies. Yellow fever outbreak—Disaster Relief Emergency Fund operation no. MDRGH005 [cited 2022 Mar 26]. https://reliefweb.int/report/ghana/yellow-fever-outbreak-dref-operation-n°-mdrgh005

[22] Nwaiwu AU, Musekiwa A, Tamuzi JL, Sambala EZ, Nyasulu PS. The incidence and mortality of yellow fever in Africa: a systematic review and meta-analysis. BMC Infect Dis. 2021;21:1089. 10.1186/s12879-021-06728-x 34688249 PMC8536483

[23] Bonney JHK, Osei-Kwasi M, Adiku TK, Barnor JS, Amesiya R, Kubio C, et al. Hospital-based surveillance for viral hemorrhagic fevers and hepatitides in Ghana. PLoS Negl Trop Dis. 2013;7:e2435. 10.1371/journal.pntd.0002435 24069490 PMC3777898

[24] Kwagonza L, Masiira B, Kyobe-Bosa H, Kadobera D, Atuheire EB, Lubwama B, et al. Outbreak of yellow fever in central and southwestern Uganda, February-may 2016. BMC Infect Dis. 2018;18:548. 10.1186/s12879-018-3440-y 30390621 PMC6215607

[25] Johansson MA, Vasconcelos PFC, Staples JE. The whole iceberg: estimating the incidence of yellow fever virus infection from the number of severe cases. Trans R Soc Trop Med Hyg. 2014;108:482–7. 10.1093/trstmh/tru092 24980556 PMC4632853

[26] Mutebi JP, Barrett AD. The epidemiology of yellow fever in Africa. Microbes Infect. 2002;4:1459–68. 10.1016/S1286-4579(02)00028-X 12475636

[27] Barrett AD, Monath TP. Epidemiology and ecology of yellow fever virus. Adv Virus Res. 2003;61:291–315. 10.1016/S0065-3527(03)61007-9 14714435

[28] de Souza RP, Foster PG, Sallum MA, Coimbra TL, Maeda AY, Silveira VR, et al. Detection of a new yellow fever virus lineage within the South American genotype I in Brazil. J Med Virol. 2010;82:175–85. 10.1002/jmv.21606 19950229

[29] Mutebi JP, Wang H, Li L, Bryant JE, Barrett AD. Phylogenetic and evolutionary relationships among yellow fever virus isolates in Africa. J Virol. 2001;75:6999–7008. 10.1128/JVI.75.15.6999-7008.2001 11435580 PMC114428

[30] von Lindern JJ, Aroner S, Barrett ND, Wicker JA, Davis CT, Barrett ADT. Genome analysis and phylogenetic relationships between east, central and west African isolates of Yellow fever virus. J Gen Virol. 2006;87:895–907. 10.1099/vir.0.81236-0 16528039

